# Gestational age, birthweight, and maternal factors associated with neonatal mortality in the United States

**DOI:** 10.3389/fpubh.2026.1840829

**Published:** 2026-08-12

**Authors:** Lin Wang, Xiaoling Shen, Qingjun Li, Lihong Hao

**Affiliations:** 1 Department of Neonatal Internal Medicine, Tianjin Children’s Hospital (Children’s Hospital, Tianjin University), Tianjin, China; 2 Department of Vascular Diseases, Affiliated Hospital of Gansu University of Traditional Chinese Medicine, Lanzhou, China

**Keywords:** birth weight, gestational age, maternal risk factors, neonatal mortality, population-based study, prenatal care

## Abstract

**Background:**

Neonatal mortality remains a key indicator of maternal and infant health and continues to show substantial variation across demographic and clinical groups in the United States. A clearer understanding of nonlinear associations and effect modification may help characterize epidemiologic patterns of neonatal mortality and inform perinatal prevention efforts.

**Methods:**

We conducted a population-based retrospective cohort study using the U.S. Linked Birth–Infant Death Database from 2015 to 2020. Neonatal mortality was defined as death occurring from day 0 through day 27 after live birth, inclusive. The study included 22,184,639 live births, among which 77,523 neonatal deaths were identified. Multivariable logistic regression was used to estimate adjusted odds ratios and 95% confidence intervals. Restricted cubic splines were used to characterize nonlinear associations, and subgroup analyses were conducted to evaluate potential effect heterogeneity.

**Results:**

Among 22,184,639 live births, 77,523 neonatal deaths occurred, corresponding to a neonatal mortality rate of 0.35%. Preterm birth was strongly associated with neonatal mortality (aOR, 4.81; 95% CI, 4.62–5.04), as was low birthweight (aOR, 5.21; 95% CI, 5.04–5.45). Late or absent prenatal care was associated with higher odds of neonatal mortality (aOR, 1.70; 95% CI, 1.60–1.82), as were non-Hispanic Black race/ethnicity (aOR, 1.45; 95% CI, 1.38–1.53), maternal smoking (aOR, 1.35; 95% CI, 1.28–1.43), hypertensive disorders of pregnancy (aOR, 1.22; 95% CI, 1.15–1.31), and male infant sex (aOR, 1.12; 95% CI, 1.08–1.16). Restricted cubic spline analyses showed nonlinear associations for both gestational age and birthweight, with the lowest mortality odds near 39–40 weeks and approximately 3,200–3,600 g.

**Conclusion:**

In this nationwide observational cohort, gestational age, birthweight, and several maternal and infant characteristics were associated with neonatal mortality. The identified nonlinear patterns and subgroup differences may help characterize population-level mortality gradients and identify groups with comparatively elevated risk, although causal inferences cannot be made.

## 1 Introduction

Neonatal mortality remains a critical public health challenge and a key indicator of maternal–infant health worldwide (1). Despite substantial improvements in obstetric and neonatal care over recent decades, the United States continues to experience neonatal mortality rates that exceed those of many other high-income countries (2). Most neonatal deaths occur within the first week of life and are strongly associated with complications of prematurity, low birthweight, and perinatal conditions that may be preventable with optimized maternal care (3, 4). Understanding population-level determinants of neonatal mortality is therefore essential for informing targeted interventions and improving outcomes.

Gestational age and birthweight are among the strongest associations of neonatal survival (5). Infants born preterm or with low birthweight face markedly higher risks of respiratory insufficiency, infection, intraventricular hemorrhage, and metabolic instability—conditions that collectively contribute to early neonatal death (6–9). Yet, despite their importance, the dose–response relationships between these continuous measures and mortality risk have not been fully clarified on a national scale. Prior studies have typically relied on categorical classifications (e.g., preterm vs. term; low birthweight vs. normal birthweight), potentially obscuring nonlinear associations that may be important for understanding population-level gradients in neonatal mortality (10). Restricted cubic spline modeling offers a flexible approach to delineate these gradients but has been underutilized in neonatal epidemiology.

Beyond infant characteristics, maternal demographic and behavioral factors—including age, race/ethnicity, educational attainment, parity, smoking and alcohol use, and prenatal care—also play critical roles in shaping neonatal outcomes (11–13). However, previous investigations often examined these predictors in isolation or in limited regional samples, leaving gaps in our understanding of how multiple maternal risk factors jointly influence neonatal survival within the contemporary U.S. population. Additionally, whether the effects of gestational age on neonatal mortality differ across maternal subgroups (e.g., age categories, parity levels, prenatal care adequacy) remains uncertain.

The Linked Birth–Infant Death (LBID) database provides a unique opportunity to address these questions because it offers nationwide coverage, standardized birth-certificate information, and linkage between live-birth and infant-death records. Previous U.S. studies have established strong gestational age– and birthweight-specific gradients in neonatal mortality; however, several influential analyses were based on earlier birth cohorts, relied primarily on predefined exposure categories, or focused on selected high-risk gestational-age groups. Contemporary national reports also commonly present descriptive mortality rates across broad gestational-age or demographic categories rather than jointly modeling the continuous shapes of the gestational-age and birthweight associations. Consequently, the nonlinear associations of both exposures and their relationship with multiple maternal and infant characteristics remain incompletely characterized in a contemporary nationwide U.S. population.

Therefore, using linked records for more than 22 million U.S. live births from 2015 to 2020, we aimed to quantify the associations of maternal, pregnancy-related, and infant characteristics with neonatal mortality; characterize the continuous nonlinear associations of gestational age and birthweight using restricted cubic splines; and evaluate variation in these associations across clinically relevant maternal and infant subgroups. By combining contemporary nationwide data, flexible nonlinear modeling, mutually adjusted sensitivity analyses, and subgroup evaluation within a single analytical framework, this study was designed to provide a more detailed characterization of population-level neonatal mortality gradients than conventional categorical analyses alone.

## 2 Methods

### 2.1 Study design and data source

This population-based retrospective cohort study used the U.S. Linked Birth–Infant Death Data Files for the calendar years 2015 through 2020, compiled by the National Center for Health Statistics. These files link information from live-birth certificates with corresponding infant death records and include maternal demographic characteristics, pregnancy and delivery information, infant characteristics, and mortality outcomes. Biologically implausible birthweight–gestational age combinations were identified using the gestational-age-specific birthweight plausibility criteria developed by Alexander et al. Records with birthweights below or above the corresponding plausible limits for the reported gestational age were excluded.

Multiple births and infants with recorded congenital anomalies were retained in the primary cohort because the study aimed to characterize neonatal mortality across the complete population of live births. The study was reported in accordance with the STROBE guidelines.

### 2.2 Exposure variables

#### 2.2.1 Gestational age

Gestational age (GA), obtained from the obstetric estimate, served as a primary exposure and was analyzed as both a continuous variable and categorical variable. Categories included:

- <34 weeks.
- 34–36 weeks.
- 37–38 weeks.
- 39–40 weeks (reference).
- ≥41 weeks.

#### 2.2.2 Birthweight

Birthweight was evaluated continuously and categorically:

- <2,500 g.
- 2,500–3,999 g.
- ≥4,000 g.

Both GA and birthweight were modeled as continuous predictors using restricted cubic splines to examine nonlinear associations with neonatal mortality.

### 2.3 Outcome

The primary outcome was neonatal mortality, defined as death occurring from day 0 through day 27 after live birth, inclusive, corresponding to death before 28 completed days of life. Infants who survived through the first 27 days of life were classified as neonatal survivors.

Covariates were selected based on clinical relevance and prior literature. Maternal factors included age, race/ethnicity, educational attainment, parity, smoking during pregnancy, gestational hypertension, diabetes (defined as either pregestational or gestational diabetes), and adequacy of prenatal care. Infant sex was also included. Gestational age and birthweight were analyzed as key exposure variables using both categorical and continuous forms, with restricted cubic spline models applied to evaluate nonlinear associations.

### 2.4 Statistical analyses

Statistical analyses were performed using R version 4.3.2. Descriptive statistics were calculated using available observations for each variable. Missing data were handled using complete-case analysis for each multivariable model; records missing the outcome, exposure, or any covariate included in a specific model were excluded from that analysis. No statistical imputation was performed. Multivariable binary logistic regression models were used to estimate adjusted odds ratios (aORs) and 95% confidence intervals (CIs) for neonatal mortality. Logistic regression was selected because neonatal mortality was defined as a binary event occurring within the fixed period from day 0 through day 27 after live birth. Covariates were selected *a priori* on the basis of clinical relevance and previous literature. Covariate inclusion was not based on univariable statistical significance, and no automated stepwise variable-selection procedure was used. The prespecified adjustment set included maternal age, race/ethnicity, educational attainment, parity, smoking during pregnancy, alcohol use during pregnancy, adequacy of prenatal care, diabetes, hypertensive disorders of pregnancy, mode of delivery, and infant sex.

For the primary gestational-age analysis, birthweight was not included in the adjustment set. For the primary birthweight analysis, the same covariates were included, but gestational age was not included. A mutually adjusted sensitivity model included both gestational age and birthweight together with all prespecified covariates. The correlation between gestational age and birthweight was evaluated using the Pearson correlation coefficient. Multicollinearity in the mutually adjusted model was assessed using variance inflation factors, with values below 5 considered to indicate the absence of serious multicollinearity. A multiplicative gestational age × birthweight interaction was additionally evaluated by adding a product term to the mutually adjusted model, with birthweight scaled per 500-g increase.

Restricted cubic spline models with four knots placed at the 5th, 35th, 65th, and 95th percentiles of the exposure distribution were used to characterize nonlinear associations of gestational age and birthweight with neonatal mortality. The spline models used the same exposure-specific adjustment sets as the corresponding regression models. Nonlinearity was assessed using likelihood-ratio tests comparing models containing the spline terms with otherwise identical models containing only a linear exposure term.

Exploratory subgroup analyses of the association between preterm birth (<37 vs. ≥37 weeks) and neonatal mortality were conducted according to maternal age (<20, 20–34, or ≥35 years), infant sex, birthweight (<2,500, 2,500–3,999, or ≥4,000 g), parity, and prenatal-care adequacy. Multiplicative interaction terms between preterm birth and each stratification variable were added separately to the corresponding multivariable models, and *p* values for interaction were reported. These analyses were considered exploratory. Model discrimination was evaluated using the area under the receiver operating characteristic curve. A two-sided *p* value <0.05 was considered statistically significant. No sampling weights were required because the data were derived from nationwide vital registration records. Record-linkage weights were not applied because the analyses used the birth cohort linked files, for which the NCHS does not recommend application of the linkage weight.

## 3 Results

### 3.1 Study population

**Figure 1:**
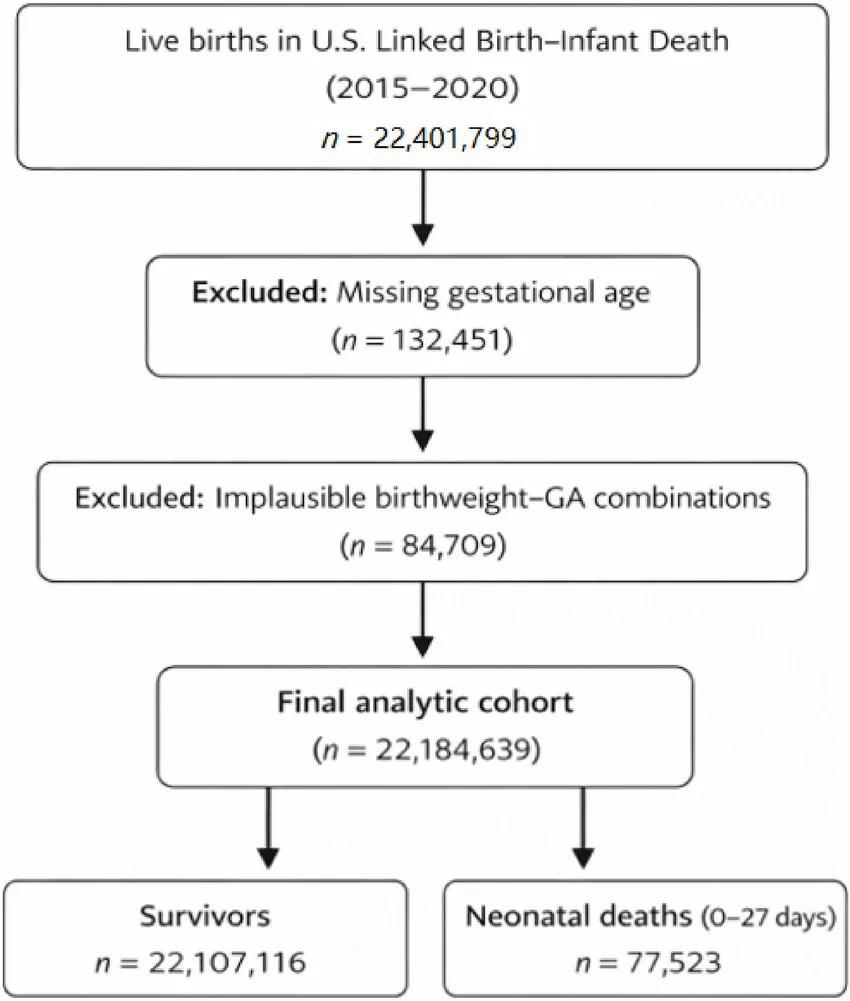
Study flowchart for inclusion and exclusion criteria. Multiple births and infants with recorded congenital anomalies were retained in the analytic cohort.

During 2015–2020, 22,401,799 live births were identified in the U.S. Linked Birth–Infant Death Data Files. We excluded 132,451 records with missing gestational age and 84,709 records with biologically implausible gestational age–birthweight combinations. Multiple births and infants with recorded congenital anomalies were retained. The final analytic cohort therefore included 22,184,639 live births, of which 77,523 infants died from day 0 through day 27 after live birth and 22,107,116 survived through day 27 (Figure 1).

Table 1 summarizes the maternal and neonatal characteristics according to neonatal survival status. All listed characteristics differed statistically between survivors and infants who died during the neonatal period (all *p* < 0.001). The largest absolute differences were observed for gestational age, birthweight, preterm birth, low birthweight, and 5-min Apgar score, whereas several maternal characteristics showed smaller absolute differences despite statistical significance.

**Table 1:** Maternal and neonatal characteristics according to neonatal survival status.

Variable	Survivors (*n* = 22,107,116)	Neonatal death (*n* = 77,523)	Total	*p* value
Maternal age, years (mean ± SD)	28.6 ± 5.9	29.1 ± 6.3	28.6 ± 5.9	<0.001
Age group (%)				<0.001
<20 years	1,764,208 (8.0%)	8,112 (10.5%)	1,772,320 (8.0%)	
20–34 years	15,661,432 (70.8%)	50,214 (64.8%)	15,711,646 (70.8%)	
≥35 years	4,681,476 (21.2%)	19,197 (24.8%)	4,700,673 (21.2%)	
Race/ethnicity				<0.001
Non-Hispanic White	10,274,539 (46.5%)	31,885 (41.1%)	10,306,424 (46.4%)	
Non-Hispanic Black	3,230,977 (14.6%)	17,948 (23.2%)	3,248,925 (14.6%)	
Hispanic	6,098,312 (27.6%)	18,640 (24.0%)	6,116,952 (27.6%)	
Asian/Pacific Islander	1,142,081 (5.2%)	3,018 (3.9%)	1,145,099 (5.2%)	
Other/unknown	1,361,207 (6.2%)	6,032 (7.8%)	1,367,239 (6.2%)	
Prenatal visits, mean ± SD	11.2 ± 3.4	9.1 ± 4.8	11.2 ± 3.4	<0.001
No prenatal care (%)	182,944 (0.83%)	1,874 (2.42%)	184,818 (0.83%)	<0.001
Diabetes (pre-gestational or gestational)	1,986,435 (9.0%)	9,723 (12.5%)	1,996,158 (9.0%)	<0.001
Hypertensive disorders	1,523,698 (6.9%)	8,420 (10.9%)	1,532,118 (6.9%)	<0.001
Smoking during pregnancy	905,472 (4.1%)	4,911 (6.3%)	910,383 (4.1%)	<0.001
Pre-pregnancy BMI (mean ± SD)	27.1 ± 5.8	27.8 ± 6.1	27.1 ± 5.8	<0.001
BMI ≥ 30 (%)	6,118,982 (27.7%)	23,791 (30.7%)	6,142,773 (27.7%)	<0.001
Cesarean delivery (%)	7,033,489 (31.8%)	29,256 (37.7%)	7,062,745 (31.8%)	<0.001
Gestational age, weeks (mean ± SD)	38.5 ± 1.9	32.6 ± 4.9	38.5 ± 1.9	<0.001
Preterm birth <37 weeks (%)	1,968,818 (8.9%)	52,216 (67.3%)	2,021,034 (9.1%)	<0.001
Birthweight, g (mean ± SD)	3,278 ± 522	2024 ± 988	3,274 ± 538	<0.001
Low birthweight <2,500 g (%)	1,684,554 (7.6%)	48,334 (62.3%)	1,732,888 (7.8%)	<0.001
Male infant (%)	11,291,561 (51.1%)	41,822 (54.0%)	11,333,383 (51.1%)	<0.001
Apgar score <7 at 5 min (%)	344,201 (1.6%)	18,745 (24.2%)	362,946 (1.6%)	<0.001

Data are presented as mean ± standard deviation or number (percentage), as appropriate. Percentages may not total 100% because of rounding. SD, standard deviation.

### 3.2 Adjusted associations between maternal, pregnancy, and infant characteristics and neonatal mortality

**Figure 2:**
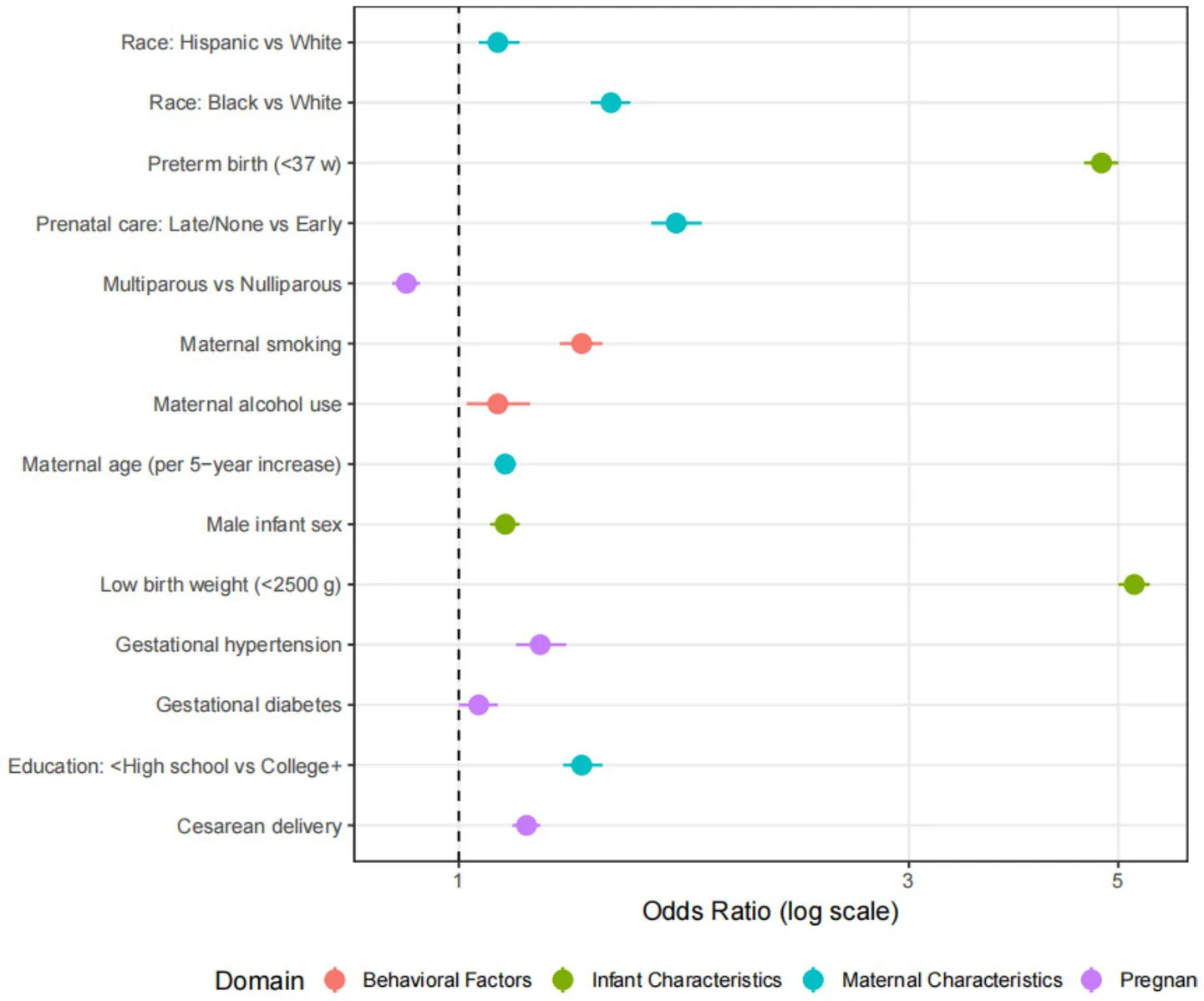
Adjusted associations of maternal, pregnancy-related, and infant characteristics with neonatal mortality. Points represent adjusted odds ratios and horizontal lines represent 95% confidence intervals derived from the multivariable logistic regression model. The corresponding numerical estimates are presented in Table 2.

The multivariable logistic regression results are presented in Table 2 and Figure 2. Compared with non-Hispanic White infants, Hispanic infants had higher odds of neonatal mortality (aOR, 1.10; 95% CI, 1.05–1.16), as did non-Hispanic Black infants (aOR, 1.45; 95% CI, 1.38–1.53). Preterm birth was strongly associated with neonatal mortality (aOR, 4.81; 95% CI, 4.62–5.04), and low birthweight was associated with similarly elevated odds (aOR, 5.21; 95% CI, 5.04–5.45).

**Table 2:** Multivariable associations of maternal, pregnancy-related, and infant characteristics with neonatal mortality.

Characteristic	Comparison	Adjusted OR (95% CI)
Race/ethnicity	Hispanic vs. non-Hispanic White	1.10 (1.05–1.16)
Race/ethnicity	Non-Hispanic Black vs. non-Hispanic White	1.45 (1.38–1.53)
Preterm birth	<37 vs. ≥37 weeks	4.81 (4.62–5.04)
Prenatal care	Late/none vs. early	1.70 (1.60–1.82)
Parity	Multiparous vs. nulliparous	0.88 (0.85–0.91)
Maternal smoking	Yes vs. no	1.35 (1.28–1.43)
Maternal alcohol use	Yes vs. no	1.10 (1.02–1.19)
Maternal age	Per 5-year increase	1.12 (1.09–1.15)
Infant sex	Male vs. female	1.12 (1.08–1.16)
Low birthweight	<2,500 vs. ≥2,500 g	5.21 (5.04–5.45)
Gestational hypertension	Yes vs. no	1.22 (1.15–1.31)
Diabetes (pregestational or gestational)	Yes vs. no	1.05 (1.00–1.10)
Maternal education	<High school vs. college or above	1.35 (1.29–1.43)
Cesarean delivery	Yes vs. no	1.18 (1.14–1.22)

Adjusted odds ratios were estimated using multivariable binary logistic regression. The fully adjusted model included maternal age, race/ethnicity, educational attainment, parity, smoking during pregnancy, alcohol use during pregnancy, prenatal care, diabetes, hypertensive disorders of pregnancy, mode of delivery, infant sex, gestational age, and birthweight. aOR, adjusted odds ratio; CI, confidence interval.

Late or absent prenatal care was associated with higher odds of neonatal mortality (aOR, 1.70; 95% CI, 1.60–1.82). Higher odds were also observed for maternal smoking during pregnancy (aOR, 1.35; 95% CI, 1.28–1.43), maternal alcohol use (aOR, 1.10; 95% CI, 1.02–1.19), each 5-year increase in maternal age (aOR, 1.12; 95% CI, 1.09–1.15), and male infant sex (aOR, 1.12; 95% CI, 1.08–1.16). Multiparity was associated with lower odds of neonatal mortality than nulliparity (aOR, 0.88; 95% CI, 0.85–0.91).

Hypertensive disorders of pregnancy (aOR, 1.22; 95% CI, 1.15–1.31), diabetes (aOR, 1.05; 95% CI, 1.00–1.10), and maternal educational attainment below high school level (aOR, 1.35; 95% CI, 1.29–1.43) were also associated with higher odds of neonatal mortality. Cesarean delivery was associated with higher adjusted odds of neonatal mortality (aOR, 1.18; 95% CI, 1.14–1.22).

The fully adjusted model showed good discrimination for neonatal mortality, with an area under the receiver operating characteristic curve of 0.934 (95% CI, 0.933–0.935).

### 3.3 Subgroup analyses

**Figure 3:**
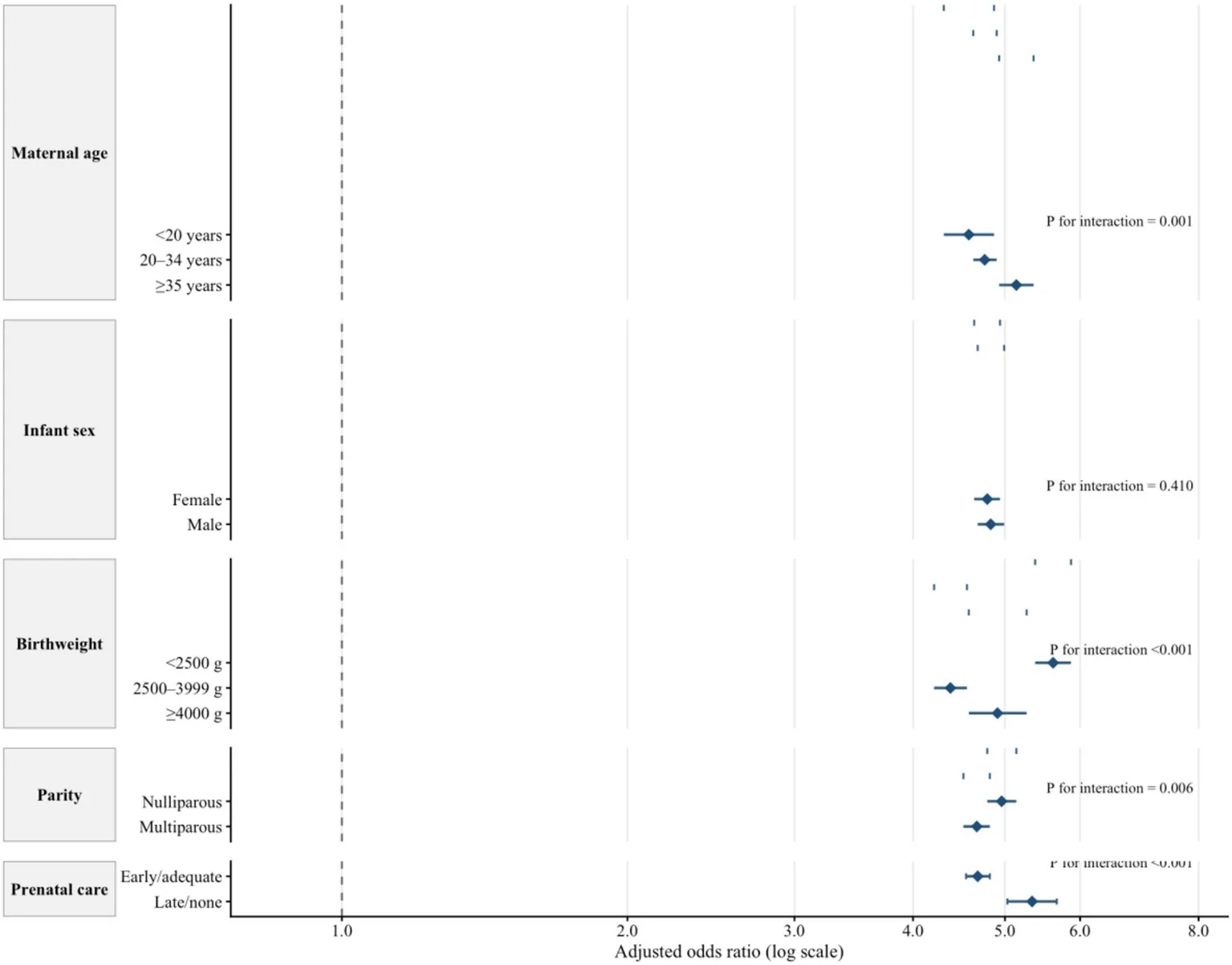
Exploratory subgroup analyses of the association between preterm birth and neonatal mortality. Adjusted odds ratios and 95% confidence intervals are shown for preterm birth (<37 vs. ≥37 weeks) across maternal age, infant sex, birthweight, parity, and prenatal-care subgroups. *p* values for interaction were obtained by including multiplicative interaction terms in the corresponding multivariable models. Numerical estimates underlying the figure are provided in Supplementary Table S1.

The association between preterm birth (<37 vs. ≥37 weeks) and neonatal mortality remained elevated across all prespecified subgroups (Figure 3). According to maternal age, the adjusted odds ratios were 4.58 (95% CI, 4.31–4.87) for mothers aged <20 years, 4.76 (95% CI, 4.63–4.90) for those aged 20–34 years, and 5.14 (95% CI, 4.93–5.36) for those aged ≥35 years (*p* for interaction = 0.001). The estimates were similar for female and male infants, with adjusted odds ratios of 4.79 (95% CI, 4.64–4.94) and 4.83 (95% CI, 4.68–4.99), respectively (*p* for interaction = 0.410).

The association varied across birthweight categories, with adjusted odds ratios of 5.62 (95% CI, 5.38–5.87) for infants weighing <2,500 g, 4.38 (95% CI, 4.21–4.56) for those weighing 2,500–3,999 g, and 4.91 (95% CI, 4.58–5.27) for those weighing ≥4,000 g (*p* for interaction <0.001). The corresponding estimates were 4.96 (95% CI, 4.79–5.14) among nulliparous mothers and 4.67 (95% CI, 4.52–4.82) among multiparous mothers (*p* for interaction = 0.006). Preterm birth also showed a stronger association among infants whose mothers received late or no prenatal care (aOR, 5.34; 95% CI, 5.03–5.67) than among those whose mothers received early or adequate prenatal care (aOR, 4.68; 95% CI, 4.55–4.82; *p* for interaction <0.001). These subgroup findings were exploratory and should be interpreted cautiously, particularly because the very large sample size may render small between-group differences statistically significant.

### 3.4 Non-linear associations of gestational age and birthweight with neonatal mortality

Gestational age and birthweight were moderately positively correlated (Pearson r = 0.62). When both variables were included in the mutually adjusted multivariable model, the maximum variance inflation factor was 1.78, indicating no evidence of serious multicollinearity. A statistically significant multiplicative interaction was observed between gestational age and birthweight (*p* for interaction <0.001). Given the very large sample size, however, this interaction was interpreted as evidence of modest effect heterogeneity rather than a large clinically meaningful interaction.

**Figure 4:**
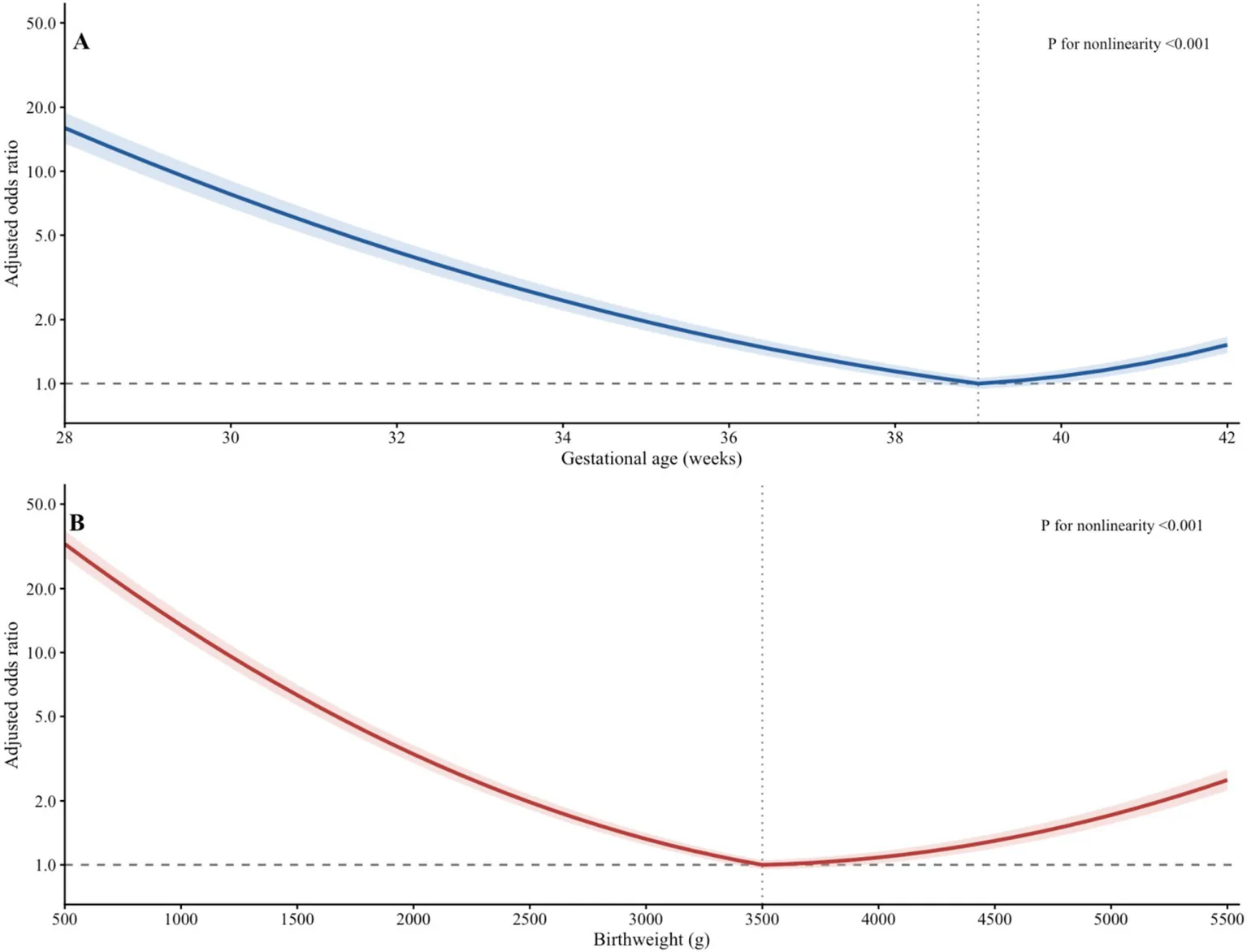
Nonlinear associations of gestational age and birthweight with neonatal mortality. Adjusted odds ratios and 95% confidence intervals were estimated using restricted cubic spline models for gestational age (**A**; reference, 39 weeks) and birthweight (**B**; reference, 3,500 g). Horizontal dashed lines indicate an odds ratio of 1.00. Both tests for nonlinearity were statistically significant (*p* < 0.001).

Restricted cubic spline analyses showed nonlinear associations of both gestational age and birthweight with neonatal mortality (both *p* for nonlinearity <0.001; Figure 4). The odds of neonatal mortality decreased sharply with increasing gestational age from approximately 32 to 37 weeks and reached their lowest level near 39–40 weeks, followed by a modest increase beyond 41 weeks. Similarly, The odds declined with increasing birthweight, reached their lowest level at approximately 3,500 g, and then gradually increased at higher birthweights. These findings support the categorical regression results and provide a more detailed description of the mortality gradients across the gestational-age and birthweight distributions.

## 4 Discussion

In this nationwide population-based cohort of more than 22 million U.S. live births, gestational age and birthweight showed the strongest associations with neonatal mortality. The associations were markedly nonlinear, with the highest mortality odds observed at very early gestational ages and very low birthweights, and the lowest odds occurring near term gestation and within an intermediate birthweight range. Maternal demographic, behavioral, and pregnancy-related characteristics, including inadequate prenatal care, smoking, hypertensive disorders of pregnancy, diabetes, and lower educational attainment, were also associated with neonatal mortality. These findings identify population groups with comparatively elevated mortality risk but should be interpreted as observational associations rather than causal effects.

The steep decline in neonatal mortality with increasing gestational age is consistent with previous U.S. linked birth–infant death analyses showing markedly higher mortality at earlier gestational ages (14, 15). This pattern is biologically plausible because earlier gestational age is associated with pulmonary immaturity, reduced surfactant production, immature immune and thermoregulatory function, and increased vulnerability to respiratory failure, infection, intraventricular hemorrhage, and metabolic instability (7). The leveling of risk near 39–40 weeks is consistent with the completion of fetal organ maturation, whereas the modest increase after 41 weeks may reflect complications associated with prolonged gestation, including placental insufficiency, meconium aspiration, or intrapartum compromise.

Birthweight showed a similarly nonlinear pattern. Very low birthweight may reflect prematurity, impaired fetal growth, or both, each of which may reduce physiological reserve during the neonatal period. At the opposite extreme, very high birthweight may be associated with maternal metabolic disorders, difficult delivery, birth trauma, or perinatal complications. The observed lowest-risk range around 3,200–3,600 g should therefore be interpreted as a population-level pattern rather than a definitive clinical threshold for individual infants.

The associations observed for maternal and pregnancy-related characteristics may reflect several overlapping pathways. Smoking during pregnancy can impair placental perfusion and fetal oxygenation and is associated with fetal growth restriction and preterm delivery (16–18). Hypertensive disorders of pregnancy may compromise uteroplacental blood flow and increase the likelihood of medically indicated preterm birth, whereas diabetes may contribute to abnormal fetal growth and delivery complications (19, 20). Inadequate prenatal care may indicate delayed recognition of maternal disease, fewer opportunities for risk monitoring, and reduced access to timely referral or delivery planning. Lower educational attainment may also act as a marker of broader socioeconomic disadvantage rather than as a direct biological determinant of neonatal mortality.

The racial/ethnic and prenatal-care differences should be interpreted within the broader U.S. healthcare and social context. Variation in insurance coverage, continuity and timing of prenatal care, neighborhood deprivation, transportation barriers, referral to maternal–fetal medicine services, and access to hospitals with advanced neonatal intensive care capabilities may contribute to differences in neonatal outcomes. Structural inequities and differential exposure to maternal morbidity may also influence the observed estimates. Accordingly, the associations involving race and ethnicity should not be interpreted as evidence of inherent biological differences between population groups. Because the public-use LBID files do not fully capture these structural and healthcare-access factors, some of the observed associations may reflect residual confounding. The positive association observed for cesarean delivery should not be interpreted as evidence that cesarean delivery itself increases neonatal mortality. Cesarean delivery is more frequently performed in pregnancies complicated by fetal distress, severe maternal disease, abnormal presentation, or anticipated preterm delivery, and the estimate is therefore likely affected by confounding by indication.

This study extends previous national analyses by integrating nonlinear modeling and subgroup analyses, thereby uncovering clinically relevant heterogeneity. For example, the consistently higher risk observed among male infants corroborates documented sex-specific differences in neonatal physiology, including delayed lung maturation, reduced surfactant synthesis, and differential inflammatory responses (21). The pronounced risk among infants with low birthweight highlights the need for enhanced perinatal surveillance and targeted intervention strategies for this group (22, 23). Moreover, the observed associations reinforce the importance of early and adequate prenatal care, a modifiable factor that remains unevenly distributed across populations and strongly predicts neonatal outcomes.

Our findings are broadly consistent with earlier U.S. evidence while extending it in several respects. Alexander et al. (14), using U.S. linked birth and infant death records from 1995–1997, demonstrated marked variation in neonatal mortality across joint gestational-age and birthweight strata and across racial and ethnic groups. More recent NCHS data have continued to show substantially higher mortality among very preterm infants and persistent differences according to maternal race and ethnicity (15). The present study confirms these established gradients in a more contemporary 2015–2020 cohort but additionally models gestational age and birthweight as continuous exposures, allowing the shapes of the associations and the lower-risk ranges to be characterized without relying exclusively on broad clinical categories. The simultaneous assessment of maternal, pregnancy-related, and infant characteristics further places these biological gradients within a broader sociodemographic and clinical context.

These interpretations are also consistent with recent U.S. studies showing that infant mortality varies according to maternal educational attainment, geographic context, and access to maternity care (24). A national analysis of more than 18 million U.S. births from 2017 to 2021 found higher infant mortality among residents of counties with no maternity-care access than among residents of counties with full access, supporting the importance of healthcare availability and structural context when interpreting individual-level associations (25, 26).

The public health implications of these findings are substantial. As neonatal mortality continues to serve as a core metric of health system performance, identifying populations at highest risk is essential for designing targeted interventions. These findings may help identify populations warranting enhanced surveillance and perinatal support. Improving access to prenatal care and addressing maternal health and behavioral factors remain important clinical and public health priorities. However, because this study was observational, the present findings do not establish that modifying any individual factor would directly reduce neonatal mortality.

This study has several strengths, including its large nationwide cohort, standardized linkage of birth and infant death records, ability to evaluate multiple maternal and infant characteristics simultaneously, and use of flexible spline models to characterize nonlinear associations. The large number of births and neonatal deaths enabled precise estimation across clinically relevant exposure ranges and supported detailed exploratory subgroup analyses. Nevertheless, several limitations should be considered. First, residual confounding remains possible despite adjustment for multiple maternal, pregnancy-related, and infant characteristics. The public-use files do not comprehensively capture household income, insurance status, neighborhood deprivation, continuity of prenatal care, referral patterns, hospital capability, or access to advanced neonatal services. Maternal conditions and behavioral factors recorded on birth certificates may also be underreported or misclassified.

Second, gestational age was based on the obstetric estimate and may be subject to measurement error, while birthweight may be affected by occasional recording or data-entry errors. A total of 217,160 records, representing approximately 0.97% of initially identified live births, were excluded because of missing gestational age or implausible birthweight–gestational age combinations. Although the excluded proportion was small, these records may have disproportionately included infants at the extremes of gestational age or birthweight; therefore, some selection bias cannot be excluded. The database also lacked detailed information on the severity of congenital anomalies, neonatal intensive care interventions, transfer patterns, and quality of neonatal care.

Third, the very large sample size may render small differences statistically significant despite limited clinical importance. In addition, because the study was based on U.S. vital-registration data, differences in population characteristics, obstetric and neonatal care practices, healthcare access, and registration systems may limit direct generalization of the absolute risks and effect estimates to other countries. Finally, the observational design precludes causal inference.

## 5 Conclusion

In this nationwide observational cohort of more than 22 million U.S. live births, gestational age, birthweight, and several maternal, sociodemographic, and pregnancy-related characteristics were associated with neonatal mortality. The nonlinear patterns identified for gestational age and birthweight provided a more detailed description of mortality-risk gradients than conventional categorical analyses alone and helped identify groups with comparatively elevated risk. Because this was an observational analysis of routinely collected data, the findings should not be interpreted as evidence of causality or as proof that modifying any individual factor would directly reduce neonatal mortality. Nevertheless, these results may inform risk stratification, future etiologic research, and the planning of targeted perinatal surveillance and support.

## Data Availability

The de-identified public-use data analyzed in this study are available from the U.S. National Center for Health Statistics Linked Birth–Infant Death Data Files. The analyses used the public-use files for 2015–2020, and no restricted-use or individually identifiable data were accessed. The statistical code used to generate the study results is available from the corresponding author upon reasonable request.
