# Supplementary material for: Gestational age, birthweight, and maternal factors associated with neonatal mortality in the United States
Source: Front Public Health. 2026 Aug 12;14:1840829. doi: 10.3389/fpubh.2026.1840829 (PMC13506870; doi:10.3389/fpubh.2026.1840829)
Supplement: Supplementary file 1 [file Table_1.docx]

| Supplementary Table S1. Numerical estimates for subgroup analyses of the association between preterm birth and neonatal mortality | | | | |
| --- | --- | --- | --- | --- |
| **Stratification variable** | **Subgroup** | **aOR** | **95% CI** | **P for interaction** |
| Maternal age | <20 years | 4.58 | 4.31–4.87 | 0.001 |
|  | 20–34 years | 4.76 | 4.63–4.90 |  |
|  | ≥35 years | 5.14 | 4.93–5.36 |  |
| Infant sex | Female | 4.79 | 4.64–4.94 | 0.410 |
|  | Male | 4.83 | 4.68–4.99 |  |
| Birthweight | <2500 g | 5.62 | 5.38–5.87 | <0.001 |
|  | 2500–3999 g | 4.38 | 4.21–4.56 |  |
|  | ≥4000 g | 4.91 | 4.58–5.27 |  |
| Parity | Nulliparous | 4.96 | 4.79–5.14 | 0.006 |
|  | Multiparous | 4.67 | 4.52–4.82 |  |
| Prenatal care | Early/adequate | 4.68 | 4.55–4.82 | <0.001 |
|  | Late/none | 5.34 | 5.03–5.67 |  |
| Note. Adjusted odds ratios represent the association between preterm birth (<37 vs. ≥37 weeks) and neonatal mortality within each subgroup. Models were adjusted for the prespecified maternal, pregnancy-related, and infant covariates, excluding the corresponding stratification variable where appropriate. P values for interaction were obtained by adding multiplicative interaction terms between preterm birth and each stratification variable to separate multivariable logistic regression models. aOR, adjusted odds ratio; CI, confidence interval. | | | | |
